# Extracts and Abstracts

**Published:** 1888-12

**Authors:** 


### EXTRACTS AND ABSTRACTS.

---

### WHEN AND HOW SHOULD DIGITALIS BE PRESCRIBED?

Dr. Henri Huchard, the distinguished clinician of Paris, has recently written a *brochure* in answer to these questions, from which we abstract the following:

One of the most important things in connection with the administration of digitalis is the cumulative action of the drug; and the following rule should be adhered to. Since the doses of digitalis add up, as it were, the drug should be prescribed in decreasing doses, and its administration should not be continued in large doses for more than four or five days. Beside the cumulation of action, there is also cumulation of dose, which Gubler defines as follows: Cumulative action is the storing of the drug in an organ (the stomach or intestine), cumulative dosage is the storing of the drug in the organism. From this must be deduced the practical conclusion that drugs that have cumulative action should be given, in so far as possible, in liquid form.

Huchard says that he very rarely has any of the unpleasant effects that are so often seen from the administration of digitalis, and that the reason is that he never violates the rules that should govern the administration of the drug. The disagreeable effects of the drug do not appear while œdema persists. While digitalis cures asystolism, it may cause a kind of toxic asystolism. Among its disagreeable effects may be mentioned a pseudo-angina, which must be distinguished from precordial anxiety, and from true anginous attacks in subjects of angina, provoked by abuse of digitalis. The most important of these effects, however, are those produced on the side of the digestive tract. The precocious vomiting caused by digitalis is due to the topical action of the drug on the gastric mucous membrane; the later vomiting comes on in about twenty-four or forty-eight hours after the administration of digitalis in large doses, and is caused by toxic action. The numerous bad effects ascribed to digitalis may dismay the practitioner, and cause him to deprive himself of one of the most useful of drugs. Much can be learned, however, from a careful reading of Huchard's answer to the question.

*When should digitalis be prescribed?* In answering this question Huchard takes up *first*, cardiac affections, and discusses the administration of digitalis according to the period of the heart disease.

Digitalis should not be given in the early phases of what Huchard calls arterial cardiopathies. For a long time he has insisted upon the capital distinction between valvular and vascular or arterial cardiopathies. The latter are nothing else than the localization of the arterio-sclerosis on the heart; they are characterized, during the greater part of their evolution, by a more or less considerable increase of arterial tension, which is the cause, not the effect, of the sclerosis. Now, in the first periods of arterio-sclerosis of the heart may be recognized all the symptoms of hypersystolism just described, and digitalis should be given, if at all, only with the greatest caution, since by increasing vascular pressure it may lead to cerebral hæmorrhages, and by it, also, the nocturnal palpitations are increased, dyspnœa becomes more intense, and anginous attacks may be provoked.

At an advanced period of cardiopathies, one often finds albumen in greater or less quantity in the urine. This is not an absolute contra-indication to the use of digitalis. The dangers of the administration of active medicines in renal affections have been much exaggerated, though there are certainly some dangers. But they demand prudence, not the doing away with drugs. That digitalis can be safely used in these cases is probably due to the fact that it is not eliminated by the kidneys, as was shown by Lafon some two years ago. While albuminuria is not positive contra-indication to the use of digitalis, therefore, it should be used with caution in these cases, in moderate doses for two or three days, and its action should be carefully watched. Used on these principles it may diminish or cause to disappear certain forms of albuminuria, when it is of cardiac origin, or produced by passive congestion of the kidneys, in the absence of any sclerous or other lesion.

In giving digitalis in valvular lesions, the seat of the lesion is sometimes of importance in indicating the drug. In aortic stenosis, for example, we know that the cardiac contractions and the pulse are slow. Then why should we give a drug that will slow them still more? By doing this we expose the patients to the dangers of digitalism. In aortic insufficiency the diastolic rest of the heart is already long enough; digitalis makes it longer, and increases the already high arterial tension caused by the disease. Why add to the injurious effects of the heart disease? In mitral insufficiency it should be prescribed only in cases of great cardiac irregularity, or of confirmed hyposystolism. In pure mitral stenosis it often causes bad effects. It should not be forgotten that mitral and aortic stenosis are the two cardiopathies that may remain latent for a long time, and require treatment late. In aortic affections, and especially in Corrigan's disease, digitalis is often contra-indicated for a long time. It raises arterial tension in an affection in which this is already exaggerated; by increasing the suddenness of the systole it gives to the blood-waves violent and repeated oscillations, which, added to the high tension, may cause cerebral hæmorrhage; it prolongs the already augmented diastolic period; finally, it contributes, by its vaso-constrictor action, toward increasing the visceral and peripheric anæmia caused by the disease. In aortic affections more than in any other, is it wrong to localize the lesion at the aortic valve, and then to take no account of the localization in the treatment.

But, says Huchard, the knowledge of the orifice affected is of secondary importance only for the indications for the drug. The therapeutic indications must be found in the cardiac muscle, as has been admirably expressed by Stokes. The situation of the cardiac souffle, its intensity and its absence, furnish no indications for the administration of digitalis; it is the state of the heart muscle and of the vessels, their feebleness (cardio-vascular asthenia) and asystolism, that demand digitalis. There exists in all cardiac affections a period intermediate between hypersystolism and asystolism, which Huchard calls hyposystolism. Suppose we have a patient in whom some months ago there were all the signs of normal or even exaggerated compensation; the precordial shock was strong, vibrating, and well limited, the apex beat slightly lowered, urine normal in quantity, no trace of visceral congestion, and no œdema of the limbs. This patient now comes with slight perimalleolar œdema in the evening, becomes breathless easily, and has palpitations and a sensation of fullness of the chest. Examination shows slight congestion of the liver, which is painful on pressure, and pulmonary hyperæmia and œdema; the cardiac contraction is soft, unequal, and irregular, the impulse more extended, more diffuse, less sensible, the apex displaced, and cardiac dullness increased transversely; the first sound of the heart is more or less dulled or weakened, the second a little loud in the region of the pulmonary orifice and to the right of the sternum. The pulse is feeble and undulating, the jugulars swollen and prominent, and the urine scanty. In this case there should be no hesitation about prescribing digitalis, for there are three capital indications—feebleness of cardiac contractility, lowering of arterial and increase of venous pressure, and scanty urine, coexisting with peripheral œdema or visceral congestion.

The urine furnishes indications for the administration of digitalis; if it becomes uratic, and falls below 800, 500, or to 200 grammes a day, digitalis should be given. In giving digitalis in heart affections we should examine the urine as carefully as we do the thermometer in fevers.

In either asystolism or hyposystolism there may be three causes of error in regard to the contra-indications of digitalis: 1. The cardiac beats may be tumultuous and violent, the impulse of the heart becoming energetic for a few moments; but examination a few moments afterward may show that the energetic systoles are followed by weak or aborted and precipitate ones, constituting the cardiataxic asystolism of Gubler. Digitalis should here be prescribed as a tonic and a regulator. 2. We may have a patient in whom the dilatation of the cardiac cavities strikes against the thin and emaciated chest walls, giving the false sensation of energetic and violent beats. Here the clinical error is doubled if a therapeutic error be made by withholding digitalis on account of supposed exaggerated compensation. 3. In asystolism the dilated right heart is in immediate relation with the thoracic wall; if it lie close to the diaphragm the beats may be communicated to the whole epigastric region. The extent of these beats is no indication of their force; they will be found feeble, undulating, and scarcely appreciable, and digitalis should be prescribed.

To recapitulate with regard to valvular lesions: Digitalis is useless in the period of eusystolism, when the lesion is compensated; it is injurious in the hypersystolic period, when the compensation is exaggerated; it is efficacious in the hyposystolic or period of transient asystolism, when the cardiac muscle and the vessels are suffering from asthenia, and when there are œdemas, visceral congestions, dropsies, and the heart beats softly and feebly; in the period of definite asystolism, or of amyocardia, when the cardiac muscle is profoundly degenerated, digitalis is sometimes useful; it may be useless, or it may be injurious. It is in these cases that caffeine in large doses sometimes gives such signally good results.

What of the use of digitalis in palpitations and tachycardia? Arterial hypertension is not always a contra-indication to the use of the drug. In a certain state of cardiac ataxia digitalis is a true regulator of the heart before being an excitor of arterial tension and before being a tonic to the heart. Let us take the case of a mitral patient in the hyposystolic or asystolic period. The pulse is small, miserable, soft, depressible, and irregular; the blood-waves are feeble, unequal, and insufficient; the precordial shock is scarcely appreciable, and the ventricular contractions are tremulous and abortive, constituting the flutterings of the heart that Stokes speaks of. But in the midst of these weak and debilitated systoles occur violent, tumultuous, and precipitate beats—a veritable state of cardiac ataxia. These palpitations do not always signify increased cardiac work. In palpitations in the hypersystolic period, digitalis is injurious; in the hyposystolic period it is useful.

Tachycardia is often confounded with palpitation of the heart. Digitalis is inefficient in the various forms of tachycardia, says Huchard

Cardiac arythmia may be classified as valvular arythmia, myocardiac, reflex, toxic, and nervous arythmia. Digitalis may be used in the first, the second is rebellious to its action, and the other three should have causal and hygienic treatment.

Two varieties of cardiac psuedo-asthma must be recognized—one seen in valvular cardiopathies, the other in arterial cardiopathies. In the first, digitalis is useful; in the second, the patient must have an exclusive milk diet for from two to four weeks.

In arterio-sclerosis of the heart, digitalis alone is contra-indicated. It may be combined with nitro-glycerine. Distilled water, 300 grammes; tincture of digitalis, 3 grammes; alcoholic solution of nitro-glycerine (au centième) gtt. xxx. Dose, two to six tablespoonfuls a day. For angina pectoris, digitalis is always contra-indicated. It some anginous cases it may be given, however, for the hyposystolic symptoms. In these cases, digitalis should be combined with nitro-glycerine or with iodide of sodium, as, iodide of sodium, 4 grammes; powdered digitalis, 2 grammes; glycerine, q. s. to make 40 pills. Take three or four a day for from five to ten days.

In the cardiac hypertrophy of puberty, and the menopause, digitalis may be given in sedative doses—small doses; in these cases preference should be given to arterial tension-depressors.

In nephritis and asystolism of renal origin, digitalis may be guardedly used in chronic parenchymatous nephritis. It should not be used in the early stages of interstitial nephritis; in the later stages, when the heart fails, and other symptoms indicate, the following may be used: Distilled water, 300 grammes; benzoate of soda and caffeine āā, 5 grammes. Dose, four to six spoonfuls a day.

In aneurism of the aorta Huchard used some such formula as: Distilled water, 300 grammes; iodide of sodium, 20 grammes; thebaic extract, 0.05 centig. Take from two to four or five tablespoonfuls a day before meals, in a little beer. Continue during twenty or twenty-five days every month. Or, distilled water, 300 grammes, alcoholic solution of nitro-glycerine (au centième), gtt. xxx. Dose, three to six tablespoonfuls a day. Lessen the dose if it causes headache.

In the first stages of exophthalmic goitre, digitalis is often useless, sometimes injurious. Later, when the heart is temporarily or permanently enfeebled, digitalis is indicated.

In the fever of endocarditis, and the phenomena of cardiac erethism, aconite should be combined with digitalis. Tincture of digitalis, 6 grammes; tincture of aconite root, 4 grammes. Take six drops three or four times a day. In the paralytic form of pericarditis (Jaccoud), tincture of digitalis may be used in doses of twenty to forty drops a day, or the infusion may be used.

In senile pneumonia and bronchitis, digitalis or caffeine must be used. Digitalis has some disadvantages. Tanret uses the following formula, as does Huchard: Benzoate of soda, 3 grammes; caffeine, 2.50 grammes; distilled water, 6 grammes. Make the solution warm. Inject subcutaneously a quantity containing 0.25 centig. of caffeine.

The following are some of the ways in which Huchard prescribes digitalis:

Alcohol (90°), 3 grammes, 50.

Homolle's amorphous digitaline, 0.02

centig. S.—Take ten drops—which contain one millig.

The following is for subcutaneous injection:

Homolle's amorphrous digitaline 0.10 centig.
Alcohol.... .... ........25 grammes.
Water ....................25 grammes.

Ten drops contain one millig. of digitaline. Inject deep twice a day into the muscular portion of the back.

*Beaujon's oxymel diuretique*—to give tone to the vessels and heart, act on the kidneys, and calm cardiac excitability.

Alcoholic tincture of digitalis...
Aqueous extract of ergot āā...10 grammes.
Gallic acid.................. 5 grammes.
Bromide of potassium......
Cherry-laurel water āā........30 grammes.
Sirup of cherry.............400 grammes.
Oxymel of squills...........515 grammes.

Dose, two to three spoonfuls a day in water.

*Diurectic and laxative powder:*

Powdered sulph. of potash.....
Soluble cream tartar..........
Powdered nitrate of potash āā..6 grammes.
Powdered digitalis leaves...... 1 gramme.

Make 20 powders. One, three times a day.

*Chomel's pills* (for albuminuria with anæmia):

Powdered squills...............
" digitalis āā.......5 grammes.
Porphyrized iron..............4 grammes.

Make 40 pills. Take from two to six a day.

*Diuretic and hydragogue pills:*

Digitalis......................
Squills .......................
Scammony āā ..................3 grammes.
Sirup of gum................ q. s.

Make 100 pills. Take from two to six a day.

### EXOPHTHALMIC GOITRE CURED BY TREATING THE NOSE.*

At a meeting of the Berlin Medical Society, January 18, 1888 (*Berliner Klinische Wochenschrift*, 1888, No. 6), Professor Fränkel reported a case of Basedow's disease which seemed to depend on hypertrophy of the nasal mucous membrane and was cured by cauterization.

The patient was a young man, seventeen years old. There was a considerable enlargement of the thyroid, and a pulse of 120 beats a minute. After having been treated for some time for these symptoms, he incidentally called attention to a narrowing of his nasal passages, which was so great as to interfere with his breathing. The galvano-cautery was applied to the inferior turbinated bone on the left side. This was followed by a marked diminution in the frequency of the pulse and by a disappearance of the goitre on the left side. Three weeks were allowed to elapse, during which no change was discovered in the patient. The pulse rate, though slower than before, was still abnormally fast, and no change occurred in the goitre. The right side was then cauterized in the same way as the left when, almost immediately, the pulse sank to normal and the goitre disappeared.

Professor Fränkel mentioned "one case, described by Hack in the year 1886, in which Basedow's disease had been cured by treatment of the nose." This report has called out a paper by Dr. Hopmann, (*Berliner Klinische Wochenscrift*, 42), in which he calls attention to a similar case reported by himself in 1885.

It would seem hardly necessary to trouble the reader with the theoretical points discussed in the paper, but the history of the case is so interesting that we give it entire.

"Mrs. Hamacher, forty years old, came under my treatment on the 11th of October, 1883, on account of a secretion of mucus in the throat, an inclination to hawk, together with cough and occasional dis-

*Berliner Klinische Wochenschrift, Nos. 6-42. Abstracted and translated by Dr. Lester Curtis.

charge of masses of firm secretion. At the same time she complained of an uncommonly great prostration, and weakness in the legs, palpitation, of the heart, and pain in the eyes, especially the right. The painful pressure and tension in the eyes are accompanied by discharge of tears. These symptoms have continued uninterruptedly for two years. Drying the face with a towel occasions an almost unendurable pain in the eyes, especially in the right; therefore, she has stopped touching the eyes when washing the face. She has often the appearance of motes and clouds before the eyes. At night there appear sparks. There is also right-sided pain in the forehead.

"The expression is peculiarly staring. Pressure upon the eyeballs, of which the right is especially prominent, is extremely painful. The pulse is 120 to 126. There exists rhinopharyngitis sicca, with extensive accumulation of scabs in the nose and roof of pharynx,—on the right side much more severe than on the left. On account of the trouble with the eyes, I sent the patient to the Cologne eye clinic where Dr. Samelsohn asserted the perfect soundness of the dioptric apparatus and the retina; still, he showed that on both sides, and especially on the right eyeball, Gräfe's symptom [want of correspondence of the motion of lid and eyeball when looking down] could be demonstrated in a very striking manner. The exophthalmus in connection with this; the very marked angina; and the general feeling of weakness, especially in the lower extremities, as well as the absence of all other local causes for the protrusion of the eyeballs, left no doubt that we had to do with those complex symptoms which we are accustomed to designate Basedow's disease, although there was no appreciable swelling of the thyroid gland.

"I will call attention to the fact that we have here an incomplete form of Basedow's disease, if we compare it with the complete form, which presents three symptoms—exophthalmus, rapid heart, and goitre, since in this case one of the symptoms is lacking. However, according to Von Bamberger, even two of them may be lacking. Von Bamberger says in regard to this: 'There are cases which continue for years with only the symptom of palpitation of the heart; others with only enlarged thyroid; others with only the symptoms in relation to the eyeballs—the exophthalmus depending upon the dilatation of the retro-bulbul blood vessels.'

"In my case the appearance of goggle-eyes was distinctly marked on the right side only, while it existed on the left side in a much less degree.

"As I have repeatedly had the experience that obstinate eye symptoms have improved from the instant in which a nasal affection, up to that time neglected, has been benefited by appropriate treatment, I turned my attention in the treatment of my patient to the quite marked rhinopharyngitis sicca. I have treated these cases for many years by the insertion of strips of cotton batting; indeed, I had already used this treatment for some time before Gottstein (1878) published his valuable communication on the use of tampons in ozœna. Since Gottstein's publication, I have extended this treatment to ozœna proper, where it is of more benefit than any other.

"In our patient there was no ozœna, only a very considerable atrophy of the mucous membrane, with a discharge of pus which was not fetid. As a result of the latter and a considerable enlargement of the nasal passages, the secretion dried in scabs and collected especially in the naso-pharynx and the roof of the pharynx, and was cast off with difficulty every few days. After the scabs were softened and removed, by the use of cotton for several days, there was a certain improvement in the pain in the forehead, the discharge of tears, and the painful feeling upon pressure in the right eyeball. I now found, on thorough investigation, that in the right nasal cavity there were small mucous polypi hanging between the atrophied middle turbinated bone and the ethmoidal cells, passing off probably from the latter. In the course of the next few days I removed three of these, of the average size of a coffee-bean.

"After this operation, a striking improvement of most of the symptoms was noticeable. The patient stated that the improvement followed almost immediately upon the conclusion of the operation. The pulse fell from 120 to 130 beats, to from 90 to 100. Pressure upon the right eyeball was not so painful as formerly. The general condition improved markedly. The patient felt herself far stronger and fresher than before.

"In the beginning of November, Gräfe's symptom, though still plainly evident on the right side, had disappeared from the left.

"On the 10th of November I noted 'absence of the muscæ volitantes, appearance of sparks in the dark, and pain on pressure in the right eyeball; exophthalmus disappearing; pulse, 92–100.'

"On the 20th of November, Dr. Samelsohn affirmed an equally marked improvement.

"Eight days later, Gräfe's symptom could be no longer detected on the right side, and the closure of the lids was normal; no feeling of pressure in the eyes or forehead; pulse, 88-92.

"Patient can wash and dry the face without experiencing the least unpleasant symptom. The insertion of cotton was continued.

"On the beginning of April, 1885, after a year and a half, the patient consulted me again, because she had been again troubled for some months with mucous secretion in the pharynx. She had been feeling entirely well and had begun to give up the use of the cotton; at least, she had used it quite irregularly, so the drying of the nasal and pharyngeal mucous membrane, as well as the collection of scabs, began to appear. Nevertheless, pain in the eyes had not appeared, nor an excessive secretion of tears or other difficulty in the eyes. Polypi had not returned; protrusion of the eyeballs was not present; pulse, 92.

"While she was in this general condition, I showed her to the general medical society here* on the 21st of April, 1885. After that time the patient was for a short time, in June, 1886, under my treatment. She complained then that for about six months she had been troubled with muscæ volitantes, clouds, and at night, the appearance of sparks. For eight days the power of vision had failed markedly, so that she was now nearly blind; she also felt weak and miserable. The eyes began to pain her again and to weep, and a 'wirr' occurred in her head whenever she strained her eyes in an attempt to see. The eyes are somewhat prominent, but Gräfe's symptom can not be produced plainly. Palpitation of the heart is occasionally present. Pulse, 96; no goitre. Fleeting pains in the back and extremities. No cotton has been used for eight months. She suffers much from dryness in the throat and hawks up almost every morning a mass of thick mucus. Polypi have not returned. Rhinopharyngitis sicca.

"The symptoms soon became better under the use of cotton, and it was scarcely two weeks before the patient could see quite well again.

"We have had to do, therefore, with a slight return of the eye symptoms in connection with, and dependent upon, an aggravation of the nasal trouble, which disappeared with the improvement of the latter."

### OHIO SANITARY ASSOCIATION.

### THE SIXTH ANNUAL CONVENTION AT CANTON, AND WHAT WAS DONE.

The sixth annual convention of the Ohio State Sanitary Association was held at Canton, Ohio, November 14 and 15. Among the members present were Dr. G. H. Ashmun, Cleveland; Dr. Byron Stanton, Commissioner of Health, of Cincinnati; Dr. D.

* Cologne.

E. Snyder, Scio; Professor A. W. Smith, Cleveland; Dr. C. O. Probst, Columbus; Professor Cady Staley, of the Case School of Applied Science, Cleveland; Henry Wood, Cleveland; Dr. T. Clark Miller, Massillon; Dr. R. Harvey Reed, F. C. Bodine, Dr. J. W. Craig, Mansfield; Dr. H. Hendrixon, Columbus; H. E. Beebe, Sidney; Dr. W. W. Pinnel, Frederickstown; Dr. A. T. Quinn, Wilmington; Dr. Thomas H. Hubbard, Toledo; Dr. John McCurdy, Youngstown; Dr. E. R. Eggleston, Mount Vernon; Professor E. A. Jones, Massillon; H. E. Daykin, Cleveland; Dr. J. C. Fahenstock, Piqua; Thomas W. Gordon, Georgetown; Dr. James Boles, Alliance; and Drs. Lewis Slusser, Josiah Hartzel, J. Whitny, C. N. Lyman, and others, of Canton.

President Ashmun occupied the chair, with Dr. R. Harvey Reed as secretary.

The reports of standing committees were called for, when Dr. T. CLARK MILLER, chairman of the legislative committee, stated that a great advance in the interests of sanitation had been made by the passage of a law by the Ohio Legislature compelling the organization of a board of health in every city and town containing more than five hundred inhabitants. He thought it was highly necessary that the people should be educated in sanitary matters.

Dr. D. H. BECKWITH, of Cleveland, was an ardent advocate of free speech, and his motion that all papers read before the association be immediately discussed was carried.

Dr. R. HARVEY REED then read a paper on the "Prevention of Typhoid Fever."

In the discussion which followed, Dr. G. C. ASHMUN, of Cleveland, said that typhoid fever often made its appearance under similar circumstances in which diphtheria had prevailed in the same locality the year before, and that, when typhoid fever prevailed, it was always with bad sanitary surroundings.

Dr. BYRON STANTON, of Cincinnati, thought there was no disease so easily prevented as typhoid fever, except, perhaps, small-pox. Last year the disease prevailed to an unusual degree in Cincinnati, no doubt caused by the city's water supply, which was taken from the Ohio River. As soon as the people commenced to boil the drinking water and that used for culinary purposes the affliction disappeared.

Dr. D. H. BECKWITH, of Cleveland, said: If we keep on with our germs and sanitary inspectors we will have to give up our ice-water and many of our foods for fear of them.

Dr. T. CLARK MILLER, of Massillon, said typhoid fever is simply a filth fever, and to avoid it was not to know whether it was produced by a germ or not, but to teach people to avoid all filth.

At the afternoon session the subject came up again.

Dr. REED believed that typhoid fever was propagated by a specific germ that was destroyed by boiling.

Dr. JOHN McCURDY, of Youngstown, Ohio, said that he did not believe that boiling water or cooking food alone would prevent typhoid fever, but that personal cleanliness was a large factor in its prevention.

Dr. JOSIAH HARTZEL then read a paper on "Canton's Water Supply."

In the discussion which followed, Dr. McCURDY said that he thought that artesian wells were of a very questionable character, but when a city could get a pure supply of water from an artesian well it was exceedingly fortunate. Youngstown, he said, was contemplating a filter system, but found it too expensive. They finally decided to get Pasteur filters and put them in the houses, furnishing them to the people at cost.

Dr. G. C. ASHMUN said that he had tested a large number of filters, but found it impossible to free the water of germs.

Dr. REED said that Dr. Sternberg stated that he had tested this filter for germs and found it proof against them when clean, but not when dirty.

"The Relation of the Work of the School to the Health of the Child" was the subject of a paper by Professor E. A. JONES, of Massillon. It was discussed by Dr. Eggleston, of Mount Vernon; Dr. Reed, of Mansfield; Dr. Miller, Dr. Ashmun and Dr. McCurdy.

Dr. E. R. EGGLESTON followed with a paper on "Meteorology as Related to Morbidity," which was discussed by Dr. Beckwith.

Dr. McCurdy presided at the evening session. The Hon. John F. Blake, mayor of Canton, briefly welcomed the association to the city, and President Ashmun responded on behalf of the association.

Dr. Miller, Dr. Reed, and Dr. Slusser were appointed to consider and report upon a suggestion of the president in regard to the association becoming auxiliary to the State Board of Health.

Dr. THOMAS HUBBARD, of Toledo, then read his paper on "Hot Air *vs.* Steam for the Heating and Ventilation of Dwellings," which provoked considerable discussion.

Dr. MCCURDY said that Youngstown had tried both steam and hot air, and he thought the latter the best.

Dr. ASHMUN stated that Cleveland had tried to utilize the cold air shafts for the dry-closet system, but failed for two reasons: First, that they were not high enough; and second, because they had too many air-draughts in them. Since these defects had been remedied, however, the system was working satisfactorily.

Dr. REED stated that he had studied heating and ventilation for years, and he preferred the warm air as the best and cheapest.

"Is it a good idea to put in a dry closet when there is a sewer system?" asked some one in the audience.

"If it is a school-house I would, but if a private dwelling I would use the sewer," the doctor replied.

Mr. BOUR, of Canton, opposed the system. "It fills the air with poisonous germs and the rooms with obnoxious smells."

Alexander McGregor thought the system a failure. He thought it wrong to imperil the health of the children by its use. Not only this, but it had been decided upon for a number of new buildings without those pushing the matter waiting to see whether it were true or false. He appealed to the people of Canton if the system was not a failure, and putting the system in other buildings under the circumstances was a mistake.

At the Thursday morning session Professor J. J. BURNS, principal of the Canton schools, read a very entertaining paper on "Contagion of Health." It was not the conventional scientific dissertation, but happy, brilliant, and original.

This was followed by a paper on "Sewers for Small Towns," by Professor CADY STALEY. At the conclusion of the paper the subject was discussed by Dr. McCurdy, Dr. Ashmun, Mr. Wood, Dr. Stanton, and others.

Professor ALBERT W. SMITH followed with a paper on "The Water Supply of Cleveland."

At the request of the committee having the matter in charge it was decided to postpone the consideration of the motion made in a previous session, to the effect that the association become auxiliary to the State Board of Health.

The nominating committee made its report at the opening of the afternoon session. They named the following, who were duly elected as the officers of the association for the ensuing year:

President, Dr. D. M. Beckwith, Cleveland.

Vice-Presidents, Josiah Hartzel and Dr. Lewis Slusser, Canton, and Professor E. A. Jones, Massillon.

Secretary, Dr. R. Harvey Reed, Mansfield.

Treasurer, Dr. M. J. Weaver, Dayton.

Later, the newly-elected president announced the standing committees for the year:

Executive Committee—M. J. Weaver, M. D., Dayton: W. J. Conklin, M. D., Professor J. J. Burns, Canton; H. E. Beebe, M. D., Sydney; Professor E. T. Nelson, Delaware.

Committee on Publication—R. Harvey Reed, M. D., Mansfield; J. C. Ashmun M. D., Cleveland; S. H. Brundage, M. D., Xenia.

Committee on Membership—John McCurdy, M. D., Youngstown; James Allison, Cincinnati; W. S. Cretchen, M. D., Bellefontaine; G. A. Collamore, M. D., Toledo.

Committee on Legislation—C. C. Probst, M. D., Columbus; T. Clark Miller, M. D., Massillon; L. C. Herrick, M. D., Columbus; Byron Stanton, M. D., Cincinnati; E. R. Eggleston, M. D., Mount Vernon.

"Heating and Ventilation" was the subject of a brief paper by FRANCIS C. BODINE, of Mansfield, which was read and discussed.

Dr. LEWIS SLUSSER followed with a paper on "Fraud in Dressed Beef," in which he said that much of it was unfit for use. That the cattle were frequently suffering from disease when killed, and the meat when it arrived at its destination was frequently sticky and contained accumulations of pus.

Dayton was decided upon as the next place of meeting, while the time was to be fixed by the executive committee. Some of the members desired that the meeting be held as near Columbus as possible, that the members of the State Board of Health might be able to be present. One of the members stated that the association was the oldest and only organization of the kind in the State, and at this, its sixth annual convention, one member of the State Board of Health was present. Another gentleman said that while he would like to have the members of the State Board of Health present, and desired that they be invited, he did not believe in importuning the members to attend.

Dr. Ashmun then presented Dr. Beckwith, the newly elected president, to the association. The doctor said that he esteemed it a very high compliment. He referred briefly to the career of the organization, and said that he would do all he could to make the year a profitable one to the members and the next meeting at Dayton a success.

The citizens of Canton returned a vote thanks, on motion of Mr. Hartzel, to the members for meeting at that place, and the association adjourned.—*The Sanitary News.*

---

### ARTIFICIAL AND NATURAL WATERS.

AMERICAN MINERAL SPRINGS—FRAUDULENT IMPORTATIONS AND SPURIOUS CLAIMS.—Dr. Enno Sander, of St. Louis, in *The National Bottlers' Gazette,* says of domestic and foreign waters:

The United States of North America have a remarkably diversified climate; they are blessed with a wonderful variety of the finest natural agricultural and horticultural products, and their mountains not only harbor in their bowels an enormous mineral wealth, but from their interior flow an endless variety of mineral waters, and a very large number of all sorts of the most valuable mineral springs. Our present knowledge of the entire surface of the country is but limited; but two years ago, the U. S. Geological Survey had already reported 8,843 individual springs in 2,822 localities, of which 787 had been analyzed, and 634 used as resorts for recreation and health, but only 223 commercially. They represent every variety of mineral waters, from the pure alkaline to the strongly purgative, impregnated with large quantities of saline ingredients, and thermal springs with a temperature ranging from 90° to 200° Fahr.

There are springs so pure that they contain but a small fraction of a grain of solid substances in one thousand grains of water; others which are strongly and fully impregnated with mineral ingredients of great medicinal value; while again others present, in the perfect composition of solids with gases and water, such an excellent combination as to render their taste exceedingly agreeable and refreshing. Under a careful and circumspect management they could be, and, to some extent, have already been, utilized as very popular resorts. Many of them, with beautiful surroundings and bountiful fresh air, offer to the sick or worn out a desirable spot, where body and mind may gradually recuperate from their infirmities, and the impaired faculties ultimately restored to their original health.

While it is granted that thousands and thousands of our fellow-countrymen and women avail themselves annually of the advantage offered to them in our own country, how is it that with all this bountiful wealth at home, foreign watering places are filled with, and almost sustained by, visitors from this country, and our markets annually over-flooded with an enormous quantity of European bottled waters? According to the "Statistical Trade Review" of the *National Bottlers' Gazette*, there were imported during the first six months of the present year, 1,564,322 gallons of mineral waters, or over 60,000 gallons more than during the same period of last year, and all of it was passed free of duty as "natural mineral water." What can be the cause of this remarkable phenomenon? Is the foreign product of a quality superior to our own? or are the majority of our people not aware of the excellent qualities of our own waters and the wonderful quantity of our natural wealth?

Bottled Manufactured Waters.—The average American citizen is conservative; he loves his country above all; he desires to develop its resources as fast as it can be done, and cheerfully supports every enterprise tending to this end; but it seems that he has not yet been sufficiently informed in regard to the importance of this mineral water question. The most beneficial use is made of natural mineral water at the spring itself, where all its qualities are unimpaired, and its full benefits can be enjoyed. But "many diseases do not admit of delay, and for this purpose the bottled waters are applicable. However, there is not the care used in bottling waters that should be observed. There are but few waters that are at all adapted to shipment; the gases escape, and some of the chemical ingredients are decomposed" (George E. Walton, M.D., "The Mineral Springs of the United States and Canada," 1883, p. 113). Besides these facts, it has been ascertained by careful and often repeated observations and analysis that mineral waters (especially those exhibiting a high temperature, and those containing sensitive metallic and earthy oxides, which are promptly affected by the oxygen of the atmosphere and converted into insoluble dioxides) are readily decomposed, and deposit some of their solid ingredients almost as soon as the water has issued from the earth. It is evident that in such cases the waters are not adapted to bottling, and the public should be warned against the acceptance of assurances, which always are readily made by the salesmen, that the bottled waters contain all the solid ingredients of, and are, therefore, equal to, the fresh waters of the springs. "Prompted partly by scientific interests, and partly by the desire to offer to sufferers, who live long distances from mineral springs, an opportunity of enjoying their beneficial action, efforts were made to reproduce these waters by artificial process. * * * And, in the course of time, this end has been attained with such perfection as to permit the artificial product of to-day to place itself alongside of and compete in every respect with the natural mineral waters" (Liebig's Chemical Dictionary, 1851, Vol. V., p. 320).

---

### THE CAUSATION OF COLD-WEATHER DISEASES.

Henry B. Baker, M. D., Secretary of the Michigan State Board of Health, in an attempt to explain the causation of inflammations of the air-passages, and the seasonal susceptibility to certain communicable diseases which are shown to be most prevalent in cold weather, has drawn the following conclusions:

1. That diphtheria, scarlet fever, and small-pox increase after the atmosphere is cold and dry, and decrease after the atmosphere is warm and moist.

2. That the three communicable diseases named above probably generally enter the body through the air-passages, and that the reason why they increase after the cold months is because of the greater susceptibility of the air-passages in those months; that this is the reason why the curves for these communicable diseases are found to follow the curves for influenza, tonsilitis, and bronchitis.

3. That the non-volatile salts of the blood exuded in excess into and upon the mucous surfaces of the air-passages are capable of leading to an inflammation which is called "influenza," "tonsilitis," or "bronchitis," according to the portion of the respiratory tract involved.

4. That, other things being equal, the non-volatile salts are left by evaporation on the mucous lining of the air-passages in proportion to the dryness of the air inhaled.

5. That, inasmuch as the absolute dryness of the air ordinarily depends upon its coldness, the inflammations of the air-passages should be expected to rise as they do after the cold dry weather, and fall after warm moist weather.

6. That the non-volatile salts are likely to be in excess in the blood under some conditions of diet, or non-action of the skin or kidneys through which, under normal conditions, they pass out of the body. Therefore,

7. That certain kinds of diet, or non-action of the skin or kidneys, may predispose to inflammation of the air-passages, and consequently to any communicable disease, which enters the body by way of the air-passages, to which the person may be susceptible.

8. That, aside from the cause herein assigned (non-volatile salts), no other known cause, capable of causing inflammation of the air-passages, is "present and acting" in proportion to the coldness and dryness of the atmosphere.

In connection with the foregoing, a few supposed facts, not entirely outside of the province of this paper, should be held in mind because they tend to modify the force of the evidence herein presented:

*a.* Vaccine virus (and, therefore, possibly the virus of the cold-weather communicable diseases) retains its vitality longer in cold than in warm weather.

*b.* The danger of contracting a communicable disease is probably increased by exposure to the contagium in a badly-ventilated room, and rooms are most frequently badly ventilated during the cold weather.

But neither of these two statements is known to be true so nearly in proportion to the temperature of the atmosphere as to explain the close correspondence with which the curves of these diseases follow the curves representing the temperature of the atmosphere. And since it is proved in this paper that the ordinary inflammations of the air-passages also follow the rises and falls of the atmospheric temperature, and are believed to be non-contagious, their equally close correspondence with the temperature changes can not be accounted for by the varying degrees of vitality of a virus, nor by bad ventilation, especially as they are so frequently traced to exposure to cold outdoor atmosphere.

9. That, so far as is yet proved by statistics of large numbers of cases, the strongest controlling cause of inflammatory diseases of the air-passages is exposure in a cold, dry atmosphere.

10. That, excepting inoculation and other similar exposure to the specific cause of the disease, the strongest controlling cause of the spread of those comunicable diseases which generally enter the body through the air-passages is exposure in a cold, dry atmosphere.

### THE PATHOGENESIS OF EPIDEMIC DIPHTHERIA.

Review of a recent work on the subject, considered from a histological standpoint, by Dr. M. J. Oertel, professor at the Munich University; with atlas of sixteen chromolithographic tables. Leipzig: F. C. W. Vogel. 1887. Translated from the *Correspondenz-Blatt für Schweizer Aertze*, Beilage No. 17, September 1, 1888, by Dr. James I. Tucker.

Though we have not succeeded in determining unerringly the kind of fungus, and, by its culture, in producing artificially the disease in exact conformity with its natural course, nevertheless the cause of diphtheria is a micro-organism.

This micro-organism settles itself principally in the oral cavity, penetrates the epithelium of the mucous membrane, and, increasing, produces a transformation in which springs forth a chemical poison. This ptomaïne (and after all not the fungus itself) gives rise to all the subsequent complex of symptoms on account of profound injury to cell-life.

This alkaloid diffuses itself in the epithelium and in the fluids of the tissues and cells. In response to the ovulation it causes the multiplying white blood corpuscles to take up the poison and perish. The effete material absorbed poisons new cells. This destructive process goes on in the mucous membrane of the throat, tonsils, and the lymphatic glands belonging to the primitive group.

The blood conveys the diseased cells (acted on by the chemical poison) to all parts of the body; these cells injure the vascular walls, the spleen, kidneys, and nervous centres. The same form of cell-destruction on all sides! The nuclear membrane dissolves; the nuclear substance, cellular substance, chromatin, break down and are transformed into a conglomerate mass. Globules, flakes, scales, granules, finely-pulverized, crumbling, dusty ruins is all that remains of the cells. Through an admixture of a fibrinous exudate from the blood arises a retiform, fibrous coagulum out of the ruins.

The peculiar brightness of the hyaline of degenerated albuminoid substances makes its appearance; increasing serous saturation liquifies the perishing mass. If this mass lies near the surface it breaks through and coagulates, forming the so-called "diphtheritic membrane"; if it lies deep the end is liquefaction and perfect dissolution.

Thus, from the immigration of the fungus, comes first a local deposit, and then a universal infection.

Since Oertel determines for this disease the morbid processes which take place in most diverse organs, thus enriching cellular pathology by an exhibition of cells, carrying on a struggle with the diphtheria-fungus, he has probably established a fact that similar cell-alterations may also occur in other infectious diseases (vide Ziemssen's Archiv., Bd. 42, s. 511). Therefore, his work has a significance of all the more general importance.

---

### A CASE OF COCAINE POISONING.

Reported by Dr. Friedrich Haenel (*Berliner Klinische Wochenschrift*, No. 44). Abstracted and translated by Dr. Lester Curtis.

A dentist injected 0.1125 ($1\frac{3}{4}$ grains) of cocaine in two doses into the gum of a girl nineteen years old, preparatory to the extraction of a tooth. Immediately after the extraction signs of unconsciousness appeared; then she grew very pale, fell, and was attacked with severe convulsions which were interrupted by short pauses. Nitrite of amyl and cold applications to the head were of no benefit.

"When I saw the patient she lay upon the sofa perfectly unconscious, groaning; her face was slightly cyanotic, and she did not react to any sort of irritation. The whole body, buttocks and extremities, were agitated by violent clonic convulsions. These continued for five hours, with pauses which became gradually longer and more frequent. The facial muscles were not affected by the convulsions. The pupils were moderately wide and without reaction. There was no exophthalmus. The skin felt warm and dry. The temperature in the axilla, taken at the end of this stage, was 38.2 [100.8° Fahr.] The pulse, in the beginning, could not be counted; later, it was 176 beats a minute. The frequency of breathing amounted to 44. After the cessation of the convulsions, the patient lay quiet for two hours, all the time unconscious.

"After her return to consciousness, she stated that she was perfectly sensible of the second cocaine injection, but did not remember anything after that. She was unable to stand; when she was straightened up she doubled together again; she could sit only doubled up; she was unable to raise her arms or to press the hand offered to her. She had intense photophobia, diminished sensibility of the skin, anæsthesia of the mucous membrane of the nose and oral cavity, entire loss of taste and smell, dryness and burning of the throat, thirst, severe retching, pulse 132, respiration 28. Cardalgia then occurred, at first slight, but becoming, afterward, very severe.

"In addition, retention of urine occurred for twenty-four hours, but, after the first scanty, concentrated urine was voided, the secretion became normal. There was sleeplessness for thirty hours, entire loss of appetite for four days. The other symptoms disappeared after two or three days. She could walk with trembling knees after forty hours. The cardialgia remained for six days. All symptoms finally disappeared.

"It may be mentioned, in addition, that the girl, except for a fractured bone and slight chlorosis, had always been well. She denied, positively, ever having suffered from convulsions. Unfortunately, I was unable to get any information from her parents. The heart and other internal organs were healthy, menstruation normal."

These symptoms are like those produced by the drug in the lower animals. These are lessened sensibility, unconsciousness, epileptiform convulsions, "which have a cortical origin, and are dependent on anæmia of the cortex, conditioned on vaso-motor spasm." There is enormous increase in the blood pressure and great acceleration in the heart's action, dependent on irritation of the acceleratory apparatus. Secretion from the mucous membrane and kidneys is diminished.

The largest safe dose of cocaine is stated by Lauderer to be 0.15 (¼ grain). Decker says 0.02 (3-10 grain), possibly, under special conditions, 0.03 (45-100 grain). Doses, however, of 0.1 (1½ grains) to 0.2 (3 grains) have been given without bad results, showing of course that great difference in the toleration of the drug exists. The largest dose ever given by Dr. Haenel was 0.035. This dose was followed by no unpleasant results.

The treatment for cases of cocaine poisoning has been indicated in the history of the case. Nitrite of amyl acts very well in slight cases.

---

### DISINFECTION AND DEODORIZING OF IODOFORM.

In the *Pharmaceutische Post* for September 30, 1888, Dr. R. Jaksch makes a statement, which we feel confident will be a surprise to many of our readers, that iodoform as an antiseptic and as a parasiticide is absolutely worthless, and he quotes a number of authors who have proved that iodoform scarcely interferes with the development of the various kinds of micro-organisms. The grounds, then, on which is based the wonderful success which has followed the employment of iodoform in the treatment of wounds seem to be thoroughly unknown, unless it may be that iodoform prevents the formation of ptomaïnes in the wound. To sterilize iodoform, Dr. Jaksch recommends its combination with an antiseptic, selecting one which would not be poisonous in the quantities which are usually employed as a local application to wounds. The author states that he has found that the antiseptics which themselves possess a specific odor, when combined with iodoform, not only reduce the odor of the latter, but largely lose their own odor, and by combination of iodoform with carbolic acid, thymol, naphthalin, and creolin, iodoform is both sterilized and deodorized. Of all these substances, the author gives the preference to creolin on the following grounds: *First*—It is the best antiseptic. *Second*—It is entirely free from any poisonous action on the higher organisms; as much as seven hundred and fifty grains have been given internally to dogs and horses without the production of the slightest symptom of poisoning. *Third*—Even in small amounts creolin thoroughly does away with the penetrating odor of iodoform.

Creolin-iodoform (1 to 2 per cent.) well rubbed up is a brownish powder of slight aromatic odor, soluble in alcohol and ether. When suspended in water the creolin is dissolved while the iodoform remains undissolved. Dr. Jaksch states that for a month or more he has been using creolin-iodoform in only 2 per cent. mixtures in the treatment of various wounds, ulcers, and abscesses, both in the form of a powder and as iodoform gauze, and he believes that his results are even better than would follow the use of the pure iodoform.—*The Therapeutic Gazette.*

